# Assessment of Pain in Endometriosis: A Radiologic Perspective on Disease Severity

**DOI:** 10.7759/cureus.65649

**Published:** 2024-07-29

**Authors:** Njood Alsudairy, Saad Alsudairy, Alaa Alahdal, Eman Alkarimi, Alaa Bakkari, Alaa Noorwali, Israa Kiram

**Affiliations:** 1 Department of Radiology, The Second Jeddah Health Cluster, Jeddah, SAU; 2 Department of Obstetrics and Gynecology, King Abdullah Medical Complex - Jeddah, Jeddah, SAU; 3 Department of Radiology, National Guard Hospital, Jeddah, SAU; 4 Department of Radiology, King Salman Bin Abdulaziz Medical City, Medina, SAU; 5 Department of Radiology, King Fahd Hospital, Medina, SAU; 6 Department of Radiology, King Fahd Hospital - Jeddah, Jeddah, SAU

**Keywords:** transvaginal ultrasound, visual analog scale, deep infiltrating endometriosis, magnetic resonance imaging, endometriosis

## Abstract

Background

Endometriosis is a prevalent gynecological disorder characterized by extra-uterine endometrial-like tissue, causing substantial morbidity, including chronic pelvic pain and infertility. Little is known about the correlation between imaging findings and pain severity in endometriosis.

Methods

We conducted a prospective observational study, enrolling 150 women diagnosed with endometriosis. Clinical, imaging (MRI and transvaginal ultrasound (TVUS)), and histopathological criteria were used for diagnosis. Pain severity was assessed using the Visual Analog Scale (VAS). Statistical analysis included multivariate regression to identify predictors of pain severity.

Results

Imaging revealed common sites of endometriosis involvement, predominantly ovaries (73.3%) and rectovaginal septum (40%). Deep infiltrating endometriosis (DIE) was present in 30% of patients, predominantly affecting uterosacral ligaments (66.7% of DIE cases). Patients with ovarian endometriomas and DIE exhibited significantly higher VAS scores (7.6 ± 1.5 and 8.0 ± 1.2, respectively) compared to those without (6.5 ± 1.9 and 6.9 ± 1.8, respectively). Surgical intervention led to a significant reduction in VAS scores (from 7.4 ± 1.6 to 3.2 ± 1.7, p < 0.001), correlating with reductions in lesion size and extent observed in follow-up imaging.

Conclusion

Advanced imaging techniques, particularly MRI and TVUS, play a critical role in assessing pain severity in endometriosis. Ovarian endometriomas and DIE are independent predictors of increased pain severity, guiding personalized treatment strategies. Surgical excision of lesions, particularly in cases of DIE, offers substantial pain relief and improves quality of life, emphasizing the integration of imaging in clinical decision-making for optimal endometriosis management.

## Introduction

Endometriosis is a chronic gynecological disorder characterized by the presence of endometrial-like tissue outside the uterine cavity, leading to inflammation, fibrosis, and the formation of adhesions. It affects approximately 10% of women of reproductive age and is a significant cause of chronic pelvic pain, dysmenorrhea, and infertility [1]. Despite its prevalence and impact on quality of life, the pathogenesis of endometriosis remains poorly understood, and its diagnosis and management pose considerable challenges [2].

The exact etiology of endometriosis is multifactorial, involving genetic, hormonal, immunological, and environmental factors [2-4]. The retrograde menstruation theory, which posits that endometrial cells are shed during menstruation and transported through the fallopian tubes into the peritoneal cavity, is widely accepted [2,5]. However, this theory does not fully explain the presence of endometriosis in distant locations, such as the lungs and brain, suggesting the involvement of additional mechanisms, such as hematogenous or lymphatic spread and coelomic metaplasia [2,6].

Clinically, endometriosis presents with a spectrum of symptoms, the most common being chronic pelvic pain, dysmenorrhea, dyspareunia, and infertility. The severity of symptoms does not always correlate with the extent of the disease, making clinical diagnosis challenging [6]. Imaging studies, particularly MRI and transvaginal ultrasound (TVUS), play a crucial role in the evaluation of endometriosis, providing valuable information on the location, size, and extent of lesions [7-10].

MRI is highly sensitive and specific for the diagnosis of endometriosis, particularly in detecting deep infiltrating endometriosis (DIE) and evaluating the involvement of pelvic organs. It provides detailed anatomical information and can differentiate endometriotic lesions from other pelvic pathologies based on their characteristic signal intensities and enhancement patterns [7]. TVUS is a widely available, non-invasive imaging modality, that is particularly effective in identifying ovarian endometriomas and superficial endometriosis [7].

The relationship between imaging findings and clinical symptoms, especially pain, remains an area of active research. While some studies have reported a correlation between the presence of endometriotic lesions and pain severity, others have found no significant association [7,11]. This discrepancy highlights the complexity of endometriosis and the need for further studies to elucidate the underlying mechanisms linking imaging features with clinical symptoms.

The management of endometriosis involves a combination of medical and surgical approaches aimed at alleviating symptoms and improving quality of life. Hormonal therapies, such as oral contraceptives, gonadotropin-releasing hormone (GnRH) agonists, and progestins, are commonly used to suppress ovarian function and reduce endometriotic lesions [11]. Surgical intervention, typically through laparoscopy, is indicated for patients with severe symptoms, large endometriomas, or when conservative treatment fails. Surgery aims to excise or ablate endometriotic lesions, restore normal pelvic anatomy, and alleviate pain [11].

## Materials and methods

Study design and patient population

This was a prospective, observational study conducted at a tertiary care center specializing in gynecological conditions. The study enrolled 150 women diagnosed with endometriosis based on clinical, imaging, and histopathological criteria. Inclusion criteria were women aged 18-45 years, with confirmed endometriosis by histopathology, who presented with pelvic pain, dysmenorrhea, or dyspareunia. Exclusion criteria included women with other pelvic pathologies, previous pelvic surgery within the last year, or those who were pregnant.

Imaging techniques

All patients underwent pelvic MRI using a 1.5T or 3T scanner. The protocol included T1-weighted, T2-weighted, and contrast-enhanced sequences. Special attention was given to high-resolution imaging of the pelvic organs and surrounding structures. MRI was used to identify endometriosis lesions, their location, size, and the extent of DIE. Lesions were classified based on their signal characteristics and enhancement patterns.

TVUS was performed using a high-frequency transducer (5-9 MHz). The ultrasound protocol included both grayscale and Doppler imaging to evaluate the ovaries, uterus, and adnexal structures. Endometriomas were identified based on their characteristic “ground-glass” appearance. The presence of pelvic adhesions and DIE was assessed by the restricted movement of pelvic organs and direct visualization of nodules or thickening.

Pain assessment

Pain severity was measured using the Visual Analog Scale (VAS) for pain, which ranges from 0 (no pain) to 10 (worst pain imaginable). Each participant rated their pain at baseline and follow-up visits. The primary outcome was the correlation between imaging findings and VAS scores.

Treatment modalities

Surgical Treatment

Patients who opted for surgical management underwent laparoscopy or laparotomy based on the extent of the disease. The surgical procedures included excision or ablation of endometriotic lesions, adhesiolysis, and cystectomy for endometriomas. All surgical interventions were performed by experienced gynecologic surgeons.

Medical Management

Patients managed medically were prescribed hormonal therapies, including oral contraceptives, GnRH agonists, and progestins. Pain management included nonsteroidal anti-inflammatory drugs (NSAIDs) and other analgesics as needed.

Follow-up and outcome measures

All patients were followed up at 3-, 6-, and 12-months post-treatment. Imaging studies (MRI and TVUS) were repeated at 6 and 12 months to evaluate changes in lesion size and characteristics. Pain scores were recorded at each follow-up visit.

Statistical analysis

Data were analyzed using IBM SPSS Statistics for Windows, Version 25 (Released 2017; IBM Corp., Armonk, NY, USA). Continuous variables were expressed as mean ± standard deviation (SD) and categorical variables as percentages. Differences in VAS scores and imaging findings between groups were analyzed using the paired t-test and ANOVA for repeated measures. Multivariate regression analysis was conducted to identify independent predictors of pain severity. A p-value of <0.05 was considered statistically significant.

## Results

Patient demographics and clinical characteristics

The study included 150 women diagnosed with endometriosis based on histopathological examination and imaging findings. The mean age of the participants was 32.4 years (range: 18-45 years). Of these, 95 (63.3%) were premenopausal, and 55 (36.7%) were postmenopausal. The average duration of symptoms was 6.7 years (range: 1-20 years), with the most common presenting symptom being pelvic pain (n = 130, or 86.7%), followed by dysmenorrhea (n = 120, or 80%) and dyspareunia (n = 90, or 60%) (Table 1).

**Table 1:** Demographic and clinical characteristics of women with endometriosis (N = 150) Demographic and clinical characteristics of 150 women diagnosed with endometriosis, including age distribution, menopausal status, symptom duration, and prevalence of pelvic pain, dysmenorrhea, and dyspareunia. N: Number of patients

Characteristic	N (%)
Number of patients	150
Mean age (years)	32.4 (18-45)
Premenopausal	95 (63.3%)
Postmenopausal	55 (36.7%)
Average duration of symptoms (years)	6.7 (1-20)
Pelvic pain	130 (86.7%)
Dysmenorrhea	120 (80%)
Dyspareunia	90 (60%)

Imaging findings

MRI and TVUS were the primary imaging modalities used to evaluate endometriosis lesions. The most common sites of involvement were the ovaries (n = 110, or 73.3%), followed by the rectovaginal septum (n = 60, or 40%), and the bladder (n = 25, or 16.7%). DIE was identified in 45 patients (30%), with lesions predominantly located in the uterosacral ligaments (n = 30, or 66.7%) (Table 2).

**Table 2:** Anatomical distribution of endometriosis lesions identified by imaging (N = 150) Distribution of endometriosis lesions across pelvic organs based on MRI and transvaginal ultrasound findings, highlighting prevalence in the ovaries, rectovaginal septum, and bladder, with a specific focus on DIE in uterosacral ligaments. N: Number of patients; DIE: Deep infiltrating endometriosis

Site of involvement	N (%)
Ovaries	110 (73.3%)
Rectovaginal septum	60 (40%)
Bladder	25 (16.7%)
Deep infiltrating endometriosis (DIE)	45 (30%)
Uterosacral ligaments	30 (66.7% of DIE)

Pain severity and imaging correlates

Pain severity was assessed using the VAS for pain, with scores ranging from 0 (no pain) to 10 (worst pain imaginable). The mean VAS score for pelvic pain was 7.2 ± 1.8. Patients with ovarian endometriomas had significantly higher VAS scores (mean: 7.6 ± 1.5) compared to those without ovarian involvement (mean: 6.5 ± 1.9; p < 0.01). Similarly, patients with DIE had higher VAS scores (mean: 8.0 ± 1.2) than those without DIE (mean: 6.9 ± 1.8; p < 0.01) (Table 3).

**Table 3:** Correlation between imaging findings and VAS scores for pelvic pain (N = 150) Comparison of VAS scores for pelvic pain between patients with and without ovarian endometriomas and DIE, demonstrating significant associations between imaging findings and pain severity. VAS: Visual analog scale; DIE: Deep infiltrating endometriosis

Pain severity (VAS)	Mean ± SD
Overall pelvic pain	7.2 ± 1.8
Ovarian endometriomas present	7.6 ± 1.5
No ovarian involvement	6.5 ± 1.9
Deep infiltrating endometriosis	8.0 ± 1.2
No deep infiltrating endometriosis	6.9 ± 1.8

Relationship between imaging features and pain

Multivariate analysis revealed that the presence of ovarian endometriomas (β = 0.35, p < 0.01) and DIE (β = 0.42, p < 0.01) were independently associated with higher pain scores. The extent of pelvic adhesions, as determined by imaging, also correlated with increased pain severity (β = 0.28, p < 0.05). There was no significant correlation between the size of endometriotic lesions and pain severity (p = 0.15).

Impact of treatment on imaging findings and pain

Among the 100 patients who underwent surgical treatment, a significant reduction in VAS scores was observed postoperatively (preoperative mean: 7.4 ± 1.6; postoperative mean: 3.2 ± 1.7; p < 0.001). MRI and TVUS follow-ups showed a reduction in lesion size and extent in 85% of the surgically treated patients. In contrast, the 50 patients managed medically showed a modest reduction in pain scores (pre-treatment mean: 7.0 ± 1.7; post-treatment mean: 5.5 ± 1.9; p < 0.05), with imaging showing minimal changes in lesion characteristics (Table 4).

**Table 4:** Effect of surgical and medical treatment on VAS scores for pain (N = 150) Comparison of pre- and post-treatment VAS scores for pain in patients undergoing surgical versus medical management, highlighting significant pain reduction post-surgery compared to modest improvement with medical therapy. VAS: Visual analog scale

Variable	β coefficient	p-value
Ovarian endometriomas	0.35	<0.01
Deep infiltrating endometriosis	0.42	<0.01
Pelvic adhesions	0.28	<0.05
Size of lesions	0.15	0.15

Adverse events and complications

Adverse events related to surgical treatment included minor complications such as wound infection (n = 5, or 5%) and urinary tract infection (n = 3, or 3%). There were no major complications reported. In the medical management group, the most common adverse effects were gastrointestinal disturbances (n = 10, or 20%) and mood changes (n = 7, or 14%) (Table 5).

**Table 5:** Adverse events and complications associated with treatment modalities (N = 150) Incidence of adverse events and complications related to surgical and medical treatments for endometriosis, including minor complications such as wound and urinary tract infections with surgical intervention, and gastrointestinal disturbances and mood changes with medical management. N: Number of patients

Adverse events	Surgical (N)	Medical (N)
Wound infection	5 (5%)	0
Urinary tract infection	3 (3%)	0
Gastrointestinal disturbances	0	10 (20%)
Mood changes	0	7 (14%)

## Discussion

Our study underscores the significant relationship between imaging findings and the severity of pain symptoms in patients with endometriosis. This correlation emphasizes the critical role of advanced imaging techniques, particularly MRI and TVUS, in the comprehensive evaluation and management of this complex condition.

We found that the presence of ovarian endometriomas and DIE were independently associated with higher pain scores, as measured by the VAS [11]. These findings are consistent with previous studies suggesting that DIE, due to its invasive nature and tendency to involve deeper pelvic structures, is particularly associated with severe pain [12]. The high VAS scores observed in patients with ovarian endometriomas may be attributed to the mass effect and local inflammatory response elicited by these lesions.

Our study also highlighted the utility of imaging in identifying pelvic adhesions, which correlated with increased pain severity. This finding suggests that adhesions, by restricting the mobility of pelvic organs and causing traction on nerves, may contribute significantly to pain in endometriosis patients. Therefore, a detailed imaging assessment of adhesions can inform surgical planning and potentially improve pain management outcomes.

The significant reduction in VAS scores post-surgery, along with the corresponding reduction in lesion size and extent observed in follow-up imaging, underscores the efficacy of surgical intervention for symptomatic relief in endometriosis. Surgical excision of endometriotic lesions, particularly in cases of DIE and extensive adhesions, appears to provide substantial pain relief and improve patients' quality of life. These findings align with existing literature advocating for surgery in patients with severe or refractory endometriosis [13,14].

Conversely, medical management, while effective in reducing pain to a lesser extent, showed minimal changes in imaging findings [15]. This outcome highlights the limitations of hormonal therapies in altering the anatomical burden of disease, despite their role in symptom control [4,16]. Our results suggest that medical management may be more suitable for patients with less severe disease or those who are not candidates for surgery.

Our study contributes to the growing body of evidence supporting the integration of advanced imaging techniques in the diagnostic and therapeutic pathways for endometriosis. The independent association of ovarian endometriomas and DIE with higher pain scores suggests that these imaging findings can serve as biomarkers for disease severity and guide treatment decisions. Future research should explore the potential of imaging-based scoring systems to stratify patients based on disease burden and predict treatment outcomes.

While our study provides valuable insights, it has several limitations. The observational design precludes the establishment of causal relationships between imaging findings and pain severity. The sample size, although adequate for detecting significant associations, may limit the generalizability of our findings to broader populations. Furthermore, pain perception is inherently subjective and can be influenced by various psychosocial factors not accounted for in this study.

## Conclusions

This study establishes a significant correlation between imaging findings and the severity of pain symptoms in endometriosis patients, highlighting the critical role of advanced imaging techniques, such as MRI and TVUS, in the comprehensive assessment of this condition. Our findings emphasize that the presence of ovarian endometriomas and DIE are key indicators of increased pain severity, underscoring the necessity for detailed imaging evaluations to guide personalized treatment strategies. The demonstrated efficacy of surgical intervention in providing substantial pain relief and reducing lesion burden further supports its role in the management of severe cases. These insights advocate for the integration of advanced imaging into routine clinical practice to enhance diagnostic accuracy, inform therapeutic decisions, and ultimately improve patient outcomes in endometriosis management.
